# Premenarchal anorexia nervosa: clinical features, psychopharmacological interventions, and rehospitalization analysis in a 1-year follow-up, controlled study

**DOI:** 10.1007/s00431-023-04960-y

**Published:** 2023-04-13

**Authors:** Jacopo Pruccoli, Rosa Pugliano, Beatrice Pranzetti, Antonia Parmeggiani

**Affiliations:** 1 IRCCS Istituto delle Scienze Neurologiche di Bologna, Centro Regionale per i Disturbi della Nutrizione e dell’Alimentazione in Età Evolutiva, UO Neuropsichiatria dell’Età Pediatrica, Bologna, Italy; 2 Dipartimento di Scienze Mediche e Chirurgiche (DIMEC), Via Massarenti 9, 40138, Università di Bologna, Bologna, Italy

**Keywords:** Premenarchal, Prepubertal, Menarche, Anorexia nervosa, Eating disorders, Childhood

## Abstract

Premenarchal anorexia nervosa (AN) represents a specific subtype of AN, defined by an onset before the menarche in females, involving unique endocrine and prognostic features. The scarce data on this condition lack case–control and follow-up studies. This is a case–control, observational, naturalistic study, involving participants with premenarchal AN (premenarchal girls presenting to the study center newly diagnosed with AN) treated with a multidisciplinary hospital intervention, compared to postmenarchal AN individuals on clinical, endocrine, psychopathological, and treatment variables. The rate of rehospitalizations on a 1-year follow-up after discharge and respective prognostic factors were assessed with a Kaplan–Meier analysis and Cox regression model. The sample included 234 AN participants (43, 18.4% with premenarchal and 191, 81.6% with postmenarchal AN). When compared to postmenarchal, premenarchal AN individuals presented with lower depressive scores (Self-Administered Psychiatric Scales for Children and Adolescents (SAFA)) (*U* = 1387.0, *p* = 0.010) and lower luteinizing hormone (LH) levels (*U* = 3056.0, *p* = 0.009) and were less frequently treated with antidepressants (*X*^2^ = 5.927, *p* = 0.015). A significant predictive model of the risk of rehospitalization (*X*^2^ = 19.192, *p* = 0.004) identified a higher age at admission (*B* = 0.522, *p* = 0.020) and a day-hospital (vs inpatient) treatment (*B* = 3957, *p* = 0.007) as predictive factors for rehospitalization at 1-year, independent from the menarchal status.

*Conclusion*: This study reports the clinical and treatment characteristics of premenarchal AN in one of the largest samples available in the current literature. Specific clinical features and prognostic factors for rehospitalization at 1-year follow-up were identified. Future studies should longitudinally investigate treatment-dependent modifications in endocrine and psychopathological measures in this population.

**Table:** 

**What is Known:** *• Premenarchal Anorexia Nervosa (AN) is a subtype of AN characterized by its onset before menarche in females and is associated with unique endocrine and prognostic features.*	
**What is New:** *• Individuals with premenarchal AN may display specific clinical profiles, with lower depressive symptoms and luteinizing hormone levels than postmenarchal controls.*

**What is Known:**

*• Premenarchal Anorexia Nervosa (AN) is a subtype of AN characterized by its onset before menarche in females and is associated with unique endocrine and prognostic features.*

**What is New:**

*• Individuals with premenarchal AN may display specific clinical profiles, with lower depressive symptoms and luteinizing hormone levels than postmenarchal controls.*

## Introduction

Anorexia nervosa (AN) is a mental health condition currently classified among feeding and eating disorders (FED), with a point prevalence of 2.5% in females and 0.3% in males [1]. The diagnostic criteria for AN reported in the Diagnostic and Statistical Manual of Mental Disorders (DSM-5) [2] include significantly low weight, fear or persistent behavior interfering with weight gain and energy intake, and a distorted body image or lack of recognition of low weight.

Illness onset and maintenance in AN are sustained by complex psychobiological factors [3], which may include endocrine elements [4], neurotransmission [5], and psychopathology [6]. Several medical complications may occur, including cardiac, bone, metabolic, and endocrinological pathologic conditions [7]. Loss of adipocytes may lead to a condition of energy-saving implying less secretion of IGF-1, GnRH, LH, FSH, estradiol, testosterone, and progesterone, while ACTH and cortisol increase as compensation [8]. AN is most often diagnosed in adolescence [9], with prepubertal AN being a rare condition [10], representing only 5% of AN cases [11] and showing a clear relationship between incidence and increasing age [12]. In a surveillance report for the British pediatric/psychiatric care, Nicholls and colleagues documented an incidence rate for AN of 1.1 per 100 000 person-years among children less than 13 years of age [12].

Food restriction is the dominant symptom in children with AN [13] though past research suggests that they exhibit less body image distortion, probably having less cognitive ability in complex concepts [14]. Individuals with childhood AN or with onset less than one year after menarche may be at greater risk for growth retardation than those with later onset, due to altered IGF-1 secretion [15]. In addition, younger individuals are at risk for protracted amenorrhea despite weight recovery and likely greater loss of brain volume, gray matter, and white matter than adolescents [16].

Previous studies have specifically addressed the individual features of premenarchal and prepubertal AN, two subcategorizations of AN. The first research on this topic is represented by case reports, using age cutoff to define prepuberty [16,17, 18]. More recently, other studies have addressed the concept of prepubertal AN, adopting mixed clinical criteria (premenarchal patients + diagnosis before 13 years + full growth potential not achieved [19]; onset before menarche [11, 20]). More clearly, Gowers and colleagues directly reviewed the notions of “early-onset” and “prepubertal” AN with particular reference to the role of the pubertal process within the condition [21]. The authors noted that the female menarche represents a late feature in the pubertal process, thus being a poor marker for puberty and that “prepuberty” is the stage before the onset of secondary sexual characteristics. This definition has been adopted for our study. Then, the researchers reported the clinical characteristics of 30 premenarchal individuals with AN, as compared to a wide control group [21]. Premenarchal AN presented with distinguishing clinical features, such as lower premorbid weight and height, anxieties about pubertal development, dramatic abstinence, and laxative abuse [21].

Children with FED may be exposed to different and frequent treatment interventions. This is typically seen in AN, where organic comorbidities may lead to inpatient treatments. Young patients undergoing inpatient interventions for psychiatric diseases may suffer from restrictions to personal activities, insufficient space, and containment [22]. Data from the established British Pediatric Surveillance System, moreover, have documented that children with FED may receive diverse psychopharmacological interventions [12]. Current guidelines on the treatment of children and adolescents with FED report the use of these treatments (mainly antidepressants and antipsychotics) across the scientific literature, despite the acknowledged side effect profile and the limited evidence for efficacy [23].

As this concise review of the previous literature describes, despite premenarchal AN representing a condition with specific clinical features, the existing evidence in this field uses non-univocal definitions and mainly relies on small samples, with pre-DSM-5 criteria.

The primary aim of the present study is to provide naturalistic evidence on the psychopharmacological and clinical (treatment level, duration of hospitalization) interventions adopted in a sample of patients with premenarchal and postmenarchal AN. The secondary aim of this study is to describe the potential differences between individuals with premenarchal AN and postmenarchal controls on the rate of rehospitalizations after a 12-month follow-up period following hospital discharge, in relation to the formerly assessed psychopharmacological and clinical variables.

## Methods

### Study design and participants

The present paper describes a case–control, observational retrospective study. The study was conducted in the context of an observational survey documenting the use of psychopharmacological interventions in a third-level Italian Regional center for FED in Children and Adolescents and was approved by the local ethical committee (code NPI-DAPSIFA2020). During the planning and execution phases of the study, Strengthening the Reporting of Observational Studies in Epidemiology (STROBE) guidelines were observed [24]. This study in all of its phases received no funds or sponsor from any company.

The study was conducted in March 2022 by retrospectively considering consecutive patients referred to the study center between 01/01/2016 and 31/12/2020 and with at least one hospitalization for eating disorders in the same center. Hospitalization was defined as an inpatient or day-hospital (DH) treatment. The day-hospital treatment program for children and adolescents with FED is comparably structured and as intensive as inpatient treatment. The hospital intervention adopted in our center entails a multidisciplinary psychological, psychopharmacological, and nutritional program. More specifically, patients admitted to a DH treatment program received a daily assessment and multiple consultations by specialized neuropsychiatrists and psychologists, as well as multiple consultations per week by a dietitian. Organic parameters, electrocardiograms (EKG), weight measures, and blood samples were weekly assessed by specialized nurses. Nutritional intake supplements were administered when needed. During their stay in the DH program, the patient consumed assisted meals, together with specialized operators. Concurrently, the patients underwent individualized and group psychotherapy; group psychotherapy for their caregivers was performed as well. The patients admitted to an inpatient service received the same treatments here described. The main differences characterizing an inpatient (against a DH) treatment were (1) greater severity of the clinical picture; (2) the need for the patients to sleep in the hospital without parents; and (3) the potential use of enteral nutrition (nasogastric tube feeding - NGT). The decision to admit an individual to an inpatient (against a DH) service already mentioned above was based on the severity of the organic condition, compliance with treatments, and the scarce availability of the family to assist and collaborate with care or reside at a distance from our center. Despite these considerations, to preserve continuity with the family and social context of the affected individuals, patients are preferably treated in the outpatient setting of our center rather than hospitalized in our inpatient or DH services. Especially, an outpatient setting which preserves continuity with the family and social environment was preferred when treating young children, and these patients are hospitalized only when a considerable organic and psychopathological severity hinders potential outpatient interventions [25]. Thus, young children hospitalized in our center are typically complex and severe cases, for which an inpatient treatment level (rather than a DH) is required.

Finally, the hospitalizations here considered (both inpatient and DH) represented unique and single interventions. Typically, admitted patients are scheduled to receive only one between an inpatient or a DH intervention, then discharged to our outpatient level, and a transition between inpatient and DH represents a condition to be used in case of specific needs (i.e., requires intensification or consolidation of care respectively). For these reasons, the cases of patients for which, after a DH treatment, an inpatient intervention was required (or vice versa) were considered, for the purpose of this study, as rehospitalizations. Given the relevant difference between an inpatient–DH transition on one side and a full rehospitalization on the same treatment level on the other, cases of inpatient–DH transitions were reported.

All the patients included in the study received the same multidisciplinary intervention, administered by the same team, in the same center, following the same clinical international guidelines [26].

The inclusion criteria were (a) a diagnosis of AN performed according to the DSM-5 criteria [2], (b) female cisgender, and (c) acquisition of informed consent. The exclusion criterion was insufficient clinical documentation.

Given the frequent and different hospitalizations that children and adolescents with AN may experience (medical, psychiatric, FED-specific) and the different therapeutic aims of these hospitalizations, for this study, patients were considered only at their first admission to the study center, which performs FED-specific inpatient and day-hospital treatments. Then, when considering rehospitalizations, all potential hospitalization types were included, if due to AN or targeted to treat AN or its symptoms.

Premenarchal AN was diagnosed according to a standardized criterion (onset of AN before menarche), adopted in the previous study in this field [21]. Given the potential confounding factors due to the retrospective nature of this study (lack of complete clinical documentation from other services before hospitalizations; errors due to personal reporting of AN patients and their parents concerning the onset of AN and menarche) patients were included in the “premenarchal AN” group if they were girls presenting to the study center with a first diagnosis with AN before the onset of the menarche. This criterion was applied only when clinically confirmed at the time of hospital admission to our center. Thus, patients were then included in the case group (premenarchal AN) if, at the time of hospital admission, they presented with primary amenorrhea. Unclear cases (e.g., individuals with an onset of AN apparently in a premenarchal phase, then experiencing menarche during AN) were excluded from the present analyses.

We considered the first-ever FED-specific, psychiatric hospitalization for AN in our center, and the “duration of illness” comes from a standardized history-taking provided by the clinician to the parents. Participants with secondary amenorrhea or resumption of the menstrual cycle were included in the control group (postmenarchal AN). The selection of the 2 groups was performed consecutively including all the patients receiving the hospital intervention during the considered period, to provide an unbiased and naturalistic observation. Given the naturalistic nature of the study, missing data were not replaced.

### Assessment methods

The primary objective of the study was to provide naturalistic evidence on the psychopharmacological and clinical (treatment level, duration of hospitalization) interventions adopted in a sample of patients with premenarchal and postmenarchal AN. Thus, psychopharmacological treatment variables were assessed by thoroughly reviewing the dates and duration of treatment, initial and maximum dosages, any reasons for treatment interruption, and possible emerging adverse drug reactions (ADR). During hospitalization, all the patients received repeated standard laboratory exams (which included blood counts, electrolytes, coagulation, transaminases, lipid profile, and glycemia) and repeated EKG. Sex hormones (luteinizing hormone (LH); estrogen; progesterone; prolactin) were assessed at 08:00 the day after hospital admission. Sex hormones were assessed and compared between premenarchal and postmenarchal AN patients. Non-univocal data exist concerning the relationship between these hormones, the clinical features, and the outcomes of AN. In a relevant study, restoring the hypothalamic–opioid inhibitory activity involved in the secretion of LH was found to predict the return of ovulatory cycles in AN patients [27]. Other studies suggested that serum E2 and LH present no predictive value on the return of menses, in individuals with AN [28]. Given this inconclusive evidence, in our study, sex hormones were considered as potential confounders for the prognosis of premenarchal and postmenarchal patients, and their levels were compared.

Nutritional interventions were collected and coded as well, including the use of nasogastric tube feeding. The program administered was a multidisciplinary hospital intervention for AN, with individual and group psychotherapeutic interventions, involving patients and their parents.

All the included patients were assessed for FED, with a psychopathological, nutritional, and biochemical screening at admission, demographic variables (age), clinical variables (duration of untreated illness, AN subtype, comorbidity), and anthropometric variables (admission, T0 and discharge, T1%BMI, and BMI). Given the presumed difference in age between the two included groups, BMI was reported only in descriptive analyses, and body weight and its changes were assessed as %BMI. Percentage BMI is calculated as ((BMI/median BMI for age and gender) × 100). The use of %BMI in children with AN is indicated by the report Junior MARSIPAN: Management of Really Sick Patients under 18 with Anorexia Nervosa [29]. Reference values for %BMI were obtained from the World Health Organization BMI-for-age charts for girls [30].

The diagnostic process was performed by neuropsychiatrists, pediatricians, and clinical psychologists trained in the field of FED following DSM-5 diagnostic criteria [2] and was supported by the following tests, administered at hospital admission, and all validated in the Italian language for the assessment of children and adolescents with FED.

All patients received the following assessment, whose results were retained in this study:

1. All patients were assessed with the Self-Administered Psychiatric Scales for Children and Adolescents (SAFA), a validated psychometric instrument used to assess psychiatric comorbidities in children and adolescents with eating disorders [31, 32]. Four subtests were considered, assessing specific psychopathological domains: eating disorder psychopathology (SAFA-P), anxiety (SAFA-A), depression (SAFA-D); and obsessive–compulsive symptoms (SAFA-O) [31].

The diagnostic process was supported with the following tests:

2. Individuals aged 13 years or older also completed the Eating Disorders Inventory-3 (EDI-3) survey, a self-assessment questionnaire routinely used in the diagnosis of ED symptoms [33]. The results of the Eating Disorder Risk Composite (EDRC) were retained for this study.
3. Children aged < 13 years at admission received the Eating Disorders Questionnaire in Childhood (EDQ-C), an assessment schedule used to diagnose eating disorders in children aged 0–12 years [34].
4. Children and adolescents aged 13 years or older received the Beck Depression Inventory-II (BDI-II) [35], the Symptom Check List-90-R (SCL-90-R) [36, 37], and the Body Uneasiness Test (BUT) [38].

A further objective of the study was the identification of potential differences between the 2 groups as regards the occurrence of new hospitalizations for FED in the 12 months following hospital discharge.

### Statistical analysis

Descriptive analyses were provided for the entire sample and the two included groups. The significance level was set at 0.05, and all tests were two-tailed. Shapiro–Wilk’s and Levene’s tests were used to assess the normality of data distribution and homogeneity of variance. Bonferroni correction was applied for multiple comparisons. Changes in %BMI across 3 different time points (hospital admission, hospital discharge, 1-year follow-up) were assessed with a repeated measures-ANOVA. Mauchly’s test was adopted to assess sphericity, and Geisser and Greenhouse’s correction was applied in cases of violated sphericity.

The rate of rehospitalizations for the premenarchal and the postmenarchal group was calculated through the Kaplan–Meier method, and the log-rank test was performed to assess potential differences between the two groups. The Cox regression analysis model was used to estimate the hazard ratio and 95% confidence interval for the premenarchal as compared with that for the postmenarchal group. Demographic, clinical, and treatment variables significantly different between the two groups in the univariate analysis were retained in the Cox regression analysis. As for weight variables, non-corrected BMI measures, which are directly influenced by age, were not accounted for as potential predictors. The sample size was determined based on the number of individuals enrolled within the study period. All the statistical analyses were performed using JASP, version 0.16.4, and SPSS v. 26 for Windows.

## Results

### Selection of the sample

A total of 390 children and adolescents with eating disorders who accessed our center during the considered period were identified and included in the study. These included 340 children and adolescents with AN (mean age 15.9 years, *F* = 350, 92.6%). Among those, 298 met the inclusion criteria. Then, 64 patients were removed from this sample after applying exclusion criteria. A total of 234 participants met the selected criteria and were retained for the final analyses.

### Sample characteristics

Two hundred and thirty-four female children and adolescents with AN were assessed, with a mean age of 15.6 (± 2.9) years. AN subtypes were restrictive AN (ANR) (*n* = 206, 88.0%) and binge-purging AN (ANBP) (*n* = 20, 8.6%). The mean duration of hospitalization was 121.6 (± 81.9) days. At the time of admission, 191 (81.6%) patients presented with postmenarchal AN, and 43 (18.4%) patients presented with premenarchal AN. The mean admission %BMI was 70.7 (± 9.1) for participants with premenarchal AN and 73.1 (± 11.9) for those with postmenarchal AN. This difference was not statistically significant (*U* = 4597.5, *p* = 0.221). The mean discharge %BMI was 82.0 (± 9.2) for participants with premenarchal AN and 79.8 (± 12.5) for those with postmenarchal AN. Again, this difference was not statistically significant (*U* = 3428.5, *p* = 0.091).

The full characteristics of the 2 groups are reported in Table 1. When compared to participants with postmenarchal AN, those with premenarchal AN showed a lower age at admission (*U* = 7002.0, *p* < 0.001), a lower SAFA-D score (*U* = 1387.0, *p* = 0.010), lower luteinizing hormone (LH) levels (*U* = 3056.0, *p* = 0.009), and were less frequently treated with an SSRI (*X*^2^ = 5.927, *p* = 0.015). Admission and discharge non-percentage-corrected BMIs, as shown in Table 1, were significantly different between the two groups.

**Table 1:** Demographic and clinical characteristics of the sample

**Variables**	**Premenarchal AN (*n*** **= 43)**		**Postmenarchal AN (*n*** **= 191)**	***X***^2^/***U*** ***p*** **value**
**Demographics**				
Age (years)	12.9 (± 2.0)		16.2 (± 2.7)	$\boldsymbol{\underline{U=7002.0,\, p < 0.001}}$
**Clinical variables (1)**				
Duration of untreated illness (months)	11.6 (± 10.8)		16.4 (± 15.7)	*U* = 3284.5,* p* = 0.024
AN subtype/ANA	ANR = 42 (97.7%) ANBP = 1 (2.3%)		ANR = 164 (85.9%) ANBP = 19 (9.9%) ANA = 8 (4.2%)	*X*^2^ = 4.743, *p* = 0.093
Comorbidity	MDD = 2 (4.7%) OCD = 3 (7.0%)		MDD = 13 (6.8%) OCD = 13 (6.8%)	*X*^2^ = 0.272, *p* = 0.873
**Psychopathological variables (1)**				
SAFA-A	53.3 (± 13.7)		59.3 (± 11.7)	*U* = 1290.0, *p* = 0.058
SAFA-D	57.0 (± 16.7)		67.2 (± 13.4)	$\boldsymbol{\underline{U=1387.0,\, p = 0.010}}$
SAFA-O	48.1 (± 11.8)		54.3 (± 10.2)	*U* = 1345.5, *p* = 0.017
SAFA-P	51.3 (± 13.3)		57.6 (± 11.0)	*U* = 1320.0, *p* = 0.035
**Endocrine measures (1)**				
LH	0.4 (± 1.0)		2.3 (± 7.4)	$\boldsymbol{\underline{U=3056.0,\, p < 0.009}}$
Estrogen	8.9 (± 17.0)		26.3 (± 65.8)	*U* = 2789.0, *p* = 0.084
Progesterone	0.8 (± 0.5)		0.9 (± 0.8)	*U* = 2300.5, *p* = 0.222
Prolactin	16.6 (± 10.9)		14.6 (± 11.4)	*U* = 573.0, *p* = 0.474
**Treatments (2)**				
SSRI	27 (62.8%)		153 (80.1%)	$\boldsymbol{\underline{X^{2}=5.927,\;p=0.015}}$
AAP	28 (66.7%)		122 (66.7%)	*X*^2^ = 0.000, *p* = 1.000
NGT	16 (37.2%)		62 (32.5%)	*X*^2^ = 0.356, *p* = 0.551
**Hospitalization**				
Duration of hospitalization (days)	99.1 (± 60.6)		126.6 (± 85.3)	*U* = 4998.5, *p* = 0.026
Treatment level	DH = 0 (0%) Inpatient = 43 (100%)		DH = 59 (30.9%) Inpatient = 132 (69.1%)	$\boldsymbol{\underline{p < 0.001}}$
**Weight measures (1)**				
Admission %BMI	70.7 (± 9.1)		73.1 (± 11.9)	*U* = 4597.5, *p* = 0.221
Admission BMI	13.1 (± 1.6)		15.0 (± 2.1)	$\boldsymbol{\underline{U=6471.5,\, p < 0.001}}$
Discharge %BMI	82.0 (± 9.2)		79.8 (± 12.5)	*U* = 3428.5, *p* = 0.091
Discharge BMI	15.2 (± 1.2)		16.3 (± 2.2)	$\boldsymbol{\underline{U=5516.0,\, p < 0.001}}$
1-year follow-up %BMI	87.2 (± 16.5)		86.1 (± 13.9)	*U* = 228.0, *p* = 0.689
1-year follow-up BMI	16.9 (± 2.8)		27.7 (± 2.5)	*U* = 302.5, *p* = 0.234

*AAP* atypical antipsychotics, *AN* anorexia nervosa, *ANBP* anorexia nervosa, binge-purging subtype, *ANR* anorexia nervosa, restrictive subtype, *BMI* body mass index, *%BMI* percentual body mass index, *LH* luteinizing hormone, *MDD* major depressive disorder, *NGT* nasogastric tube feeding, *OCD* obsessive–compulsive disorder, *SAFA* Self-Administered Psychiatric Scales for Children and Adolescents, *SAFA-A* anxiety subtest, *SAFA-D* depression subtest, *SAFA-O* obsession subtest, *SAFA-P* eating disorders subtest.

(1) Bonferroni-corrected significance level adjusted for a number of 4 (*p* = 0.05/4 = 0.0125); (2) Bonferroni-corrected significance level adjusted for a number of 3 (*p* = 0.05/3 = 0.0167). Significant differences are marked in bold and underlined

The assessment of potential changes of %BMI across 3 time points (hospital admission, hospital discharge, 1-year follow-up) revealed no significant difference between premenarchal and postmenarchal AN (*F* = 0.0002, *p* = 0.989). A significant effect of time (independent from pre-/postmenarchal status) was documented, with %BMI significantly increasing during the follow-up (*F* = 51.8, *p* < 0.001).

### Rehospitalization analysis

**Fig. 1:**
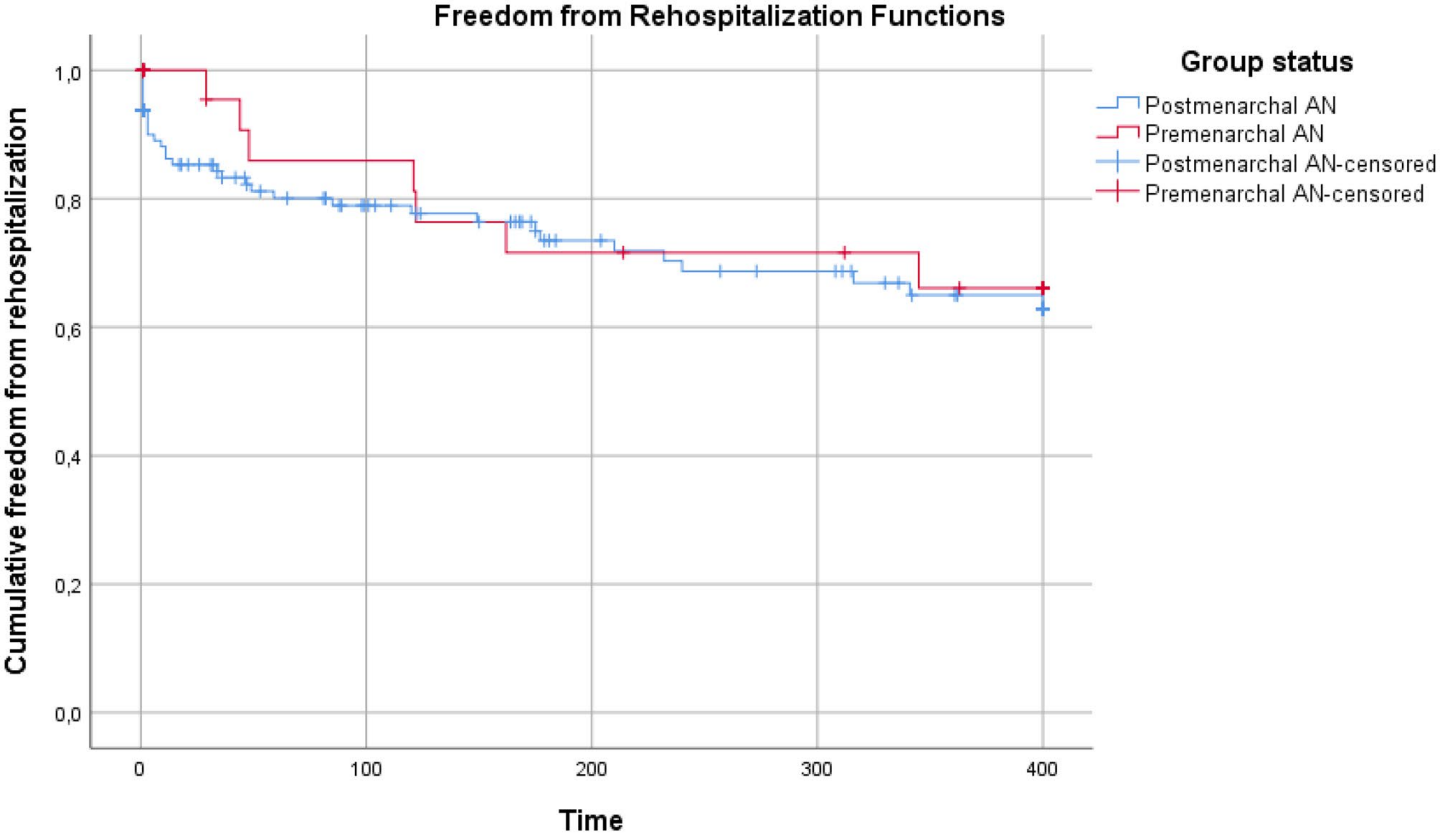
Freedom from rehospitalization analysis of the two groups. Abbreviation: AN, anorexia nervosa

Kaplan–Meier curves for premenarchal AN and postmenarchal AN groups are reported in Fig. 1. The mean time from discharge to rehospitalization was 308.4 (95% CI, 247.9–368.8) days for the premenarchal AN group and 295.5 (95% CI, 265.0–326.0) days for the postmenarchal AN group. For 12 patients, all belonging to the postmenarchal AN group, rehospitalization represented an unplanned transition between an inpatient and a DH setting (inpatient to DH: 4 cases; DH to inpatient: 8 cases).

The cumulative freedom from rehospitalization at 12 months was 66.1% (95% CI, 45.5–86.7) for the premenarchal AN group and 62.9% (95% CI, 52.1–73.7) for the postmenarchal AN group. The rate of freedom from rehospitalization at 1 year was not significantly different between premenarchal and postmenarchal AN participants (log-rank test: *X*^2^ = 0.553, *p* = 0.457).

The Cox proportional hazard model (Table 2) revealed a statistically significant predictive model of the risk of rehospitalization (*X*^2^ = 14.831, *p* = 0.020). A higher risk of rehospitalization was significantly predicted by a higher age at admission (*B* = 0.522, hazard ratio = 1686, *p* = 0.020) and by a DH treatment level (*B* = 3957, hazard ratio = 52,312, *p* = 0.007).

**Table 2:** Cox regression analysis for the included variables

	B	SE	Wald	df	Sig	Exp(B)	Lower CI	Upper CI
**Treatment level**	3957	1462	7324	1	**.007**	52,312	2978	918,840
Premenarchal AN	−.405	.900	.202	1	.653	0.667	.114	3893
**Age**	.522	.224	5431	1	**.020**	1686	1087	2616
SSRI	.066	1200	.003	1	.956	1068	.102	11,212
LH	−.014	.138	.010	1	.920	0.986	.752	1293
SAFA-D	.014	.029	.253	1	.615	1015	.959	1073

Significant predictors are marked in bold

*AN* anorexia nervosa, *LH* luteinizing hormone, *SAFA-D* Self-Administered Psychiatric Scales for Children and Adolescents, Depression subtest; *SSRI* selective serotonin reuptake inhibitors

For all reported patients, the studied initial hospitalization represented their first mental health hospitalization. Two patients (with postmenarchal AN) received 3 hospitalizations.

## Discussion

The present study aimed at reporting the clinical and endocrine features of participants with premenarchal AN, in the context of a multidisciplinary hospital intervention for FED. The sample here reported represents one of the largest samples of individuals with premenarchal AN described in the literature so far, studied for a follow-up period of 1 year. The 43 cases here presented expand the scant existing literature on premenarchal and prepubertal participants, mainly formed by case reports [16], studies in smaller samples, frequently diagnosed before the publication of DSM-5 criteria [19, 21, 39], and mixed diagnoses (76 participants with AN or an eating disorder not otherwise specified [20]).

The sample analyzed consisted of patients diagnosed exclusively with AN, so it was not possible to observe how the FED subtype (bulimia nervosa, binge eating disorder, avoidant-restrictive food intake disorder, pica, and rumination disorder) affects hospitalization time or other variables considered in this study. Despite patients with different AN subtypes (restrictive/binge-purging AN) and individuals with atypical AN being included in this study, no significant difference between the premenarchal/postmenarchal groups emerged.

The predictive model here reported for rehospitalization rates 1 year after hospital discharge identified age at admission and a DH treatment level as significant predictors of rehospitalization. No direct effect of a prepubertal/pubertal status was identified. These results are partially contradictory to those reported by Carrot and colleagues, clearly identifying premenarchal AN as a predictor of poor outcomes for AN [40]. Positive results, nonetheless, have been reported by Jaite and colleagues. Scientific literature showed that children with AN exhibited higher admission and discharge BMI percentile and reported that these clinical characteristics have been associated with better outcomes [41]. Future studies are required to examine whether these factors are associated with positive outcomes [41]. Cardinal differences in the criteria used to define participants in literature studies on childhood AN (i.e., prepubertal AN, premenarchal AN, childhood AN, early-onset AN) significantly limit the possibility for clinicians and researchers to compare the results of different studies. When interpreting the higher risk of rehospitalization for patients treated with a DH level in our study, it should be acknowledged that for 12 patients, rehospitalization represented an unplanned transition between an inpatient and a DH services. All these cases belonged to the postmenarchal AN group. Clinicians should consider that age-specific guidelines recommend the adoption of low-intensity treatment levels when dealing with children and adolescents with FED [23]. Future studies should longitudinally compare the effect of DH and inpatient treatments on premenarchal AN individuals, given the relevance of the presence and involvement of family members in the treatment of these conditions [23].

In our sample, participants with premenarchal AN presented with lower LH levels and depressive symptoms (as assessed with SAFA-D score) than those with postmenarchal AN. The finding of differences in LH levels may be linked to the different menarchal statuses of the two groups. Since LH did not show a predictive value for the risk of rehospitalization during the follow-up for our patients, this study confirms the evidence of previous research, documenting no clear predictive clinical value for LH in individuals with AN [27]. The assessment of psychiatric comorbidities in children with AN represents a major challenge in the medical literature, and the past, as well as recent research comparing the prevalence of psychiatric comorbidities, did not identify any difference between childhood and adolescent AN [42, 43]. Studies directly targeting participants with AN in childhood documented high rates (15.5%) of depressive comorbidity [44]. In our sample, moreover, participants with premenarchal AN received a SSRI less frequently than those with postmenarchal AN (62.8% vs 80.1%, respectively). Interestingly, postmenarchal AN individuals presented with higher SAFA-D (depression) scores as well. These data may be compared to those reported by Nicholls and colleagues on childhood eating disorders, documenting a relatively infrequent use of SSRIs (on 208 individuals, fluoxetine was prescribed for nine individuals, sertraline for four, and one not stated) [12]. We may hypothesize that individuals with postmenarchal AN may more frequently experience depressive symptoms and a major depressive disorder, which has a reported age at onset in the mid-20 s [45], potentially due to their higher age at admission. This may reflect in higher SSRI prescriptions. Moreover, a previous study assessing the attitudes of caregivers towards psychotropic prescriptions in children aged 12 years or less documented that despite most parents accepting psychotropic drugs for their children, if necessary, caregivers preferred to start with psychotherapy before psychopharmacology, suggesting that parental worries should be considered in this field [46]. Given the comprehensive lack of evidence in this field, further research should investigate the clinical response of depression symptoms to targeted treatment in participants with premenarchal AN.

This study has some limitations. The retrospective nature may have limited the quality of the provided data. The setting (third-level center specialized in FED in the developmental age, monocentric nature) may reduce the possibility to compare these data with those from other services. The relatively included follow-up is relatively short. Lastly, data concerning the onset of menarche and AN, in a non-longitudinal study, may have reduced reliability. Nonetheless, this study also shows some strengths. It reports one of the largest samples in the present scientific literature with premenarchal AN. Clinicians involved in the assessment and treatment of the enrolled individuals were all specialized in the field of FED in the developmental age. Lastly, the study provides data concerning multiple treatment areas, including psychopharmacological interventions, the use of NGT, and treatment level (inpatient vs DH).

## Conclusions

The present research documented the clinical, endocrine, psychopathological, and treatment features of a wide sample of participants with premenarchal AN, as compared to a group of postmenarchal AN individuals assessed in the same third-level center for FED. Premenarchal AN participants showed specific clinical characteristics, including lower depressive psychopathology, less frequent treatment with SSRI, and lower LH levels. Higher age at admission and a DH treatment level were significantly associated with an increased risk of rehospitalization at 1-year follow-up, independent from the pubertal status, as previously described in the literature [47]. Limitations of this study are a lack of information about long-term follow-up and detailed prior treatment history for FED. Further, targeted studies adopting standardized definitions are necessary for this field to report the distinct clinical features of premenarchal and postmenarchal AN and plan specific treatment protocols.

## Data Availability

The datasets used and analyzed during the study are available from the corresponding author on reasonable request.
